# Editorial: Methods in gastrointestinal cancers

**DOI:** 10.3389/fonc.2023.1193964

**Published:** 2023-04-19

**Authors:** Yue Liu, Xinyue Wang, Jingyuan Wang, Chang Wu, Wanshun Li, Qiuyue Song, Zhendong Jin

**Affiliations:** Department of Gastroenterology, Changhai Hospital, Shanghai, China

**Keywords:** detection, respectively artificial intelligence, early detection of cancer, artificial intelligence, gastrointestinal cancers

As editors of this Research Topic, it was our pleasure to review a wide range of interesting and innovative articles in the field of methods in gastrointestinal cancers. In this Editorial, we summarize the findings and perspectives regarding the application of artificial intelligence (AI) in methods in gastrointestinal cancers.

Artificial intelligence is a new technology in which researchers design and create relevant algorithms, process relevant data, and draw corresponding conclusions without human assistance. We have summarized the perspectives detailed within each of the accepted articles about AI.

First, we consider the high incidence of gastric cancer (GC), which has increased the global burden. As it is frequently diagnosed at a late stage, gastric cancer has a high mortality rate. EUS, MRI, CT, PET-CT, and other imaging examination techniques are widely used in the diagnosis, treatment, and prognosis of gastric cancer. With the development of endoscopic technology and the increasing awareness of early screening among populations, the cure rate of most early gastric cancers has been significantly improved compared with the previous period, but the survival rate of advanced gastric cancer is still very low. Thus, for gastric cancer, early detection, diagnosis, and treatment play a very important role in reducing mortality and improving survival. However, owing to some objective reasons, such as the lack of professional knowledge and experience of endoscopists, the final diagnostic accuracy is biased. At the same time, through its continuous development, the diagnostic advantages of AI are constantly expanding compared with traditional imaging techniques. Thus, by reading the literature published on this topic, we have found that convolutional neural networks and deep learning are the current hot spots of AI in gastric cancer diagnosis and differential diagnosis. Feng et al. constructed an auxiliary EGC detection system based on DCNN and compared the diagnostic abilities of DCNN and endoscopists. DCNN has good diagnostic sensitivity and rapid diagnosis characteristics, the goal is to provide solutions. In the future, the use of AI technology may help endoscopists find more cancers earlier, thus greatly reducing the general incidence in the population. Considering the great potential for the clinical application of AI, imaging examination combined with AI will continue to be a research hot spot in the future.

Second, we consider pancreatic cancer (PC), which is common in the digestive system. PC is often only found when it is advanced; therefore, it has a high fatality rate and is known as the “king of cancers”. Machine learning techniques have shown broad application potential in the diagnosis, differential diagnosis, treatment evaluation, and prediction of pancreatic diseases. For example, (1) the application of AI with malignant pancreatic tumors mainly established the machine learning model from Stephen, which integrated the free DNA fragment pattern of the whole genome, with detection sensitivity ranging from 57% to more than 99% and a specificity of 98%.^2^ This research makes the early screening and diagnosis of pancreatic malignancies possible. (Yin et al.) The technology combined with AI can shorten diagnosis time and improve diagnosis accuracy compared with traditional CT/MRI and other imaging techniques. (Qiu et al.) In terms of treating malignant pancreatic tumors, the role played by AI is mainly focused on predicting the possibility of surgery and the prognosis of patients. With the continuous development of algorithm technology, it is possible to predict postoperative complications in patients. (4) EUS-FNA/B has played an important role in the diagnosis of pancreatic malignancies. The rapid development of AI means it can be combined with EUS-FNA/B to reduce or even avoid misdiagnosis caused by the inadequate diagnostic ability of physicians in some areas with underdeveloped technologies. In the future, as algorithms continue to improve, AI may be used as a screening tool for pancreatic EUS-FNA/B specimens. (5) AI can also be used in the treatment of malignant pancreatic tumors. Based on an in-depth analysis of clinical examinations and inspection results, it is possible to carry out personalized treatment for patients with malignant pancreatic tumors.

Third, we consider rectal cancer, which has one of the highest incidences of malignant tumors in the world. Owing to the lack of awareness of patients during physical examination, rectal cancer is often diagnosed late. Even after the radical resection of rectal cancer, some patients will still develop liver metastases. Rectal cancer with liver metastasis often has a poor prognosis and a low 5-year survival rate. Owing to its incidence increasing year by year, rectal cancer has become one of the major problems that threaten human health. The liver is the most common site of distant metastasis of rectal cancer. The presence or absence of liver metastases is often a major consideration in treatment regimens. Through a retrospective study, Qiu et al. developed an XGB-based machine learning model to predict the risk of liver metastases in rectal cancer, which used a machine learning algorithm that combined seven clinical and pathological features.

It will be fascinating to observe the development of AI. Although it may face many challenges in the future, AI has shown great potential for the diagnosis and treatment of diseases, including gastrointestinal cancer.

## Author contributions

Article writing and draft manuscript preparation: YL, XYW, JYW. Article modification: CW, WSL, QYS. All authors contributed to the article and approved the submitted version.

